# Buffalo Academy of Medicine

**Published:** 1895-07

**Authors:** 


### Society Proceedings.

### BUFFALO ACADEMY OF MEDICINE.

*Meeting held October 9, 1894.*

### REPORT OF THE COMMITTEE ON HYGIENE TO INVESTIGATE THE WATER, ICE AND MILK SUPPLIES OF BUFFALO.

CHAS. G. STOCKTON, M. D., *Chairman.*

(1) Influence of our harbor and its connecting canals upon our water supply, by P. W. VANPEYMA, M. D., *Chairman of Sub-committee.*

Several months ago the committee on sanitation reported progress. A brief review of this first report naturally precedes a rehearsal of what has since been done.

The report stated that a comprehensive plan had been formulated, and that the work of the committee was as yet uncompleted. The contamination of the Niagara river from the water of the harbor and of the Buffalo creek and Hamburg canal was considered, and reference made to the facts that the harbor receives water from the Buffalo creek, the latter a receptacle of direct sewage from a portion of the city, and also in free communication, by means of the Ohio basin and Clark and Skinner slips, with the Main and Hamburg canal. The Hamburg canal being practically a continuation of the Erie canal and this again connecting with Buffalo river by means of the Commercial slip, as well as with the harbor through Peacock, Erie and Niagara slips.

The report included those of Drs. Bissell and Krauss, and of Drs. Hill and Krauss, regarding the condition of Niagara water; also that of the board of public works, stating the times when Bird Island pier inlet had been opened. It included, also, statistics obtained by Dr. Hopkins from the report of the state board of health. It referred to the cleaning of the new reservoir, and the turning off of the water supply from the public schools. The regular and frequent examination, by the health department, of the water supply was recommended; and the disinfection of the excreta of typhoid fever patients was urged.

Since the presentation of this report the committee has thought well to divide the remaining work; and as a sub-committee of one the speaker reports as follows upon the questions which were assigned to him:

An interview has been had with the city engineer regarding the feasibility of establishing a separate sewer system for surface water. The opinion of the engineer was that the expense would be altogether too great.

An effort was made to determine the population of the several districts having separate sewer systems. No tables exist giving these facts; but, based on the vote of the section, about 30,000 people live in the district sewering into the Buffalo creek and Hamburg canal.

Regarding other districts, it was found that the Bird avenue district sewer opens into the Swan street trunk sewer. The Hertel avenue sewer opens into the Erie canal; while all the rest of the region north of Scajaquada creek opens into the Niagara river about one mile below the opening of the trunk sewer. At various times in the past, attempts have been made by means of floats and the taking of levels, to determine the direction of the currents in the various slips mentioned. The observations have been so few in number, that the results are inconclusive. It is, however, settled that the currents vary, and that at times as the result of freshets, they are actually reversed. Regarding the southern part of the city, it is admitted, by those in position to know, that during freshets the sewage is forced backwards. There appears, however, to be no remedy for this, except that proposed by the board of public works, namely, to reverse the flow of sewage in this section, and then pumping it into the trunk sewer.

The report of Dr. Bissell and that of Dr. Hill, regarding the condition of the Niagara water during the preceding months, are appended.[1] It will be seen that the examinations have been regularly and frequently made, and that, on the whole, the condition of the city's water supply is satisfactory. Drs. Hill and Bissell are entitled to praise for the systematic and careful examinations which they have prosecuted. It need hardly be recommended, in conclusion, that the department of health continue its good work.

BUFFALO, October 8, 1894.

*Dr. P. W. Van Peyma :*

Dear Doctor: These analyses show that there has been but very little variation in the quality of the city water during the months covered by these reports. There has been a slight increase in albuminoid ammonia during the Summer, corresponding to the increased vegetable growth. Animal life, as shown by the microscope, has been very scanty and only such forms have been present as frequent clear running water. The water, as a whole, has been in *good* condition during the period covered by these reports.

Respectfully,

HERBERT M. HILL, *City Chemist.*

---

(2) *a*, Jubilee Water Works. *b*, Lake water and the influence of other cities on our water supply, by Wm. C. Krauss, M. D., *Chairman of sub-committee.*

JUBILEE WATER WORKS.

Between the years 1830 and 1835 a stock company was formed and a charter granted to Messrs. Gorton, Howell, Justin, *et al.*, to supply the village of Black Rock with water for domestic purposes. Black Rock at that time contained a population of 4,000 to 5,000 inhabitants, and the question of a pure water supply was an important one, as wells had been sunk fifty to seventy-five feet without obtaining any water and hundreds of dollars had been spent to no better advantage. The discovery of the springs south-

1. See table at the end of report.

east of the village, now at the corner of Auburn and Delaware avenues, led to the formation of the stock company with a franchise granted by the Legislature of the State of New York to lay pipes and to control the land lying five feet on each side of the mains, as long as these were in active use.

A bountiful supply of pure cold spring water piped into every part of the town was hailed with the greatest enthusiasm and a Black Rock jubilee was celebrated in a becoming manner, so much so that the name of the corporation was changed to the Jubilee Water Works Company. This company afterwards disposed of its interest to the Parish Tract and commissioners were elected to superintend and manage its affairs.

The water was conducted through wooden mains—the ones nearest the springs having a diameter of ten inches—to wells, better cisterns scattered throughout the village. These were about five feet in diameter and the covers were five feet below the surface. They were so arranged that when filled the supply would be cut off and no more water would enter the cistern until pumped empty. These cisterns have been gradually abandoned and filled up, until today there are perhaps thirty to thirty-five in use, the number diminishing as the water is piped into the houses or the Buffalo City water substituted. Having a fall of but fifteen and one-third feet, the water could be piped only into the first stories of the buildings.

On the annexation of Black Rock to Buffalo certain concessions were demanded and granted, one of the most important being the right to elect commissioners and to preserve the autonomy of the Jubilee Water Works. On several occasions the City of Buffalo has attempted to condemn this water supply, but chemical analysis has always shown its superiority over the Buffalo water supply.

The present condition of this water supply is as follows: Two reservoirs are situated near the corner of Auburn and Delaware avenues and one reservoir on Amherst street. But thirty to thirty-five cisterns and pumps remain and not over 250 families are supplied at present, the number diminishing with each succeeding year. These families live on Amherst, Peters, Howell and Pacific streets and the upper end of Hertel avenue. The rates per annum are $4.00 for one-story house, $5.00 for one and one-half-story house, $6.00 for two-story house and $10.00 for saloons and business places.

Houses not supplied by the city water and lying in the Jubilee

Water Works territory are obliged to pay water taxes to the Jubilee company for the use of the cistern water.

The present commissioners are Messrs. Shepard, Esser and Argus of Black Rock, and Mr. Philipbar as superintendent.

Whether or not the citizens who used the Jubilee water during the typhoid epidemic were immune from the infection, would be difficult and almost impossible to say. If during and preceding the epidemic they had abstained entirely from using or drinking Niagara water, or from coming in contact with typhoid fever patients or from using milk and produce coming from the infected district—in other words, if they and their Jubilee water had been quarantined for a sufficient time previous to the outbreak, and cases of typhoid fever had occurred in various parts of the district,—then it would be safe to say that in all probability the Jubilee water contained the typhoid bacilli. But as these conditions did not exist no definite conclusion can be drawn. It is only fair to say that the Jubilee water was less exposed to contamination, and, therefore, less liable to have been infected than the Niagara river water.

### INVESTIGATIONS OF TYPHOID FEVER OUTBREAKS DURING THE YEAR 1893 IN THE CITIES SITUATED ON LAKE ERIE.

The question whether the recent typhoid fever epidemic and the many isolated cases of typhoid occurring from time to time in this city could be attributable to the infection of Lake Erie through the sewage of the lake ports is an important one and likely to become more so as these cities increase in population. The sewage of Cleveland, Dunkirk, Toledo, Erie, Sandusky, Ashtabula and Detroit is emptied, without any treatment, into the waters of Lake Erie ; some of it in the small bays tributary to the lake, some just off shore and some ten miles out from shore. In other words, not to speak of garbage, street cleanings, etc., the excrement of nearly 1,000,000 human beings is dissolved daily in the headwaters of our natural reservoir, and not until very recently was any attempt made by individual consumers in Buffalo at purification.

Perhaps the greatest disease-producing germ in human excrement is the Eberth typhoid bacillus, a germ whose pathogenetic qualities are as universally recognized and accepted as the existence of the germ itself. The viability of this bacillus is notorious, its most favored medium being polluted water, and it is endowed by Nature to withstand the extremes of heat and cold. Of itself very motile and favored with a current of four to five miles per hour, it could very easily navigate from one end of the lake to the other, and, alas for Buffalo ! it would invariably start from the other end.

An epidemic of typhoid fever breaking out in any of the lake ports, the excreta being thrown into the sewers and thence into the lake, the danger to Buffalo might and would be very great. Desirous of ascertaining the condition of affairs, letters were accordingly addressed to the health officers of these cities asking for the number of typhoid fever cases occurring during the year 1893, the number of deaths and the disposition of the excrement of such patients. Replies were received from three cities only, and these are herewith appended :

THE CITY OF CLEVELAND, O.,
DEPARTMENT OF POLICE, PUBLIC HEALTH DIVISION,
March 22, 1894.

Wm. C. Krauss, M. D., Buffalo, N. Y. : The number of reported cases of typhoid fever, 1893, were 281. Deaths from said cause, 153.

Respectfully,
E. L. DORAN, *Sec'y.*

Estimated population is 325,000.

CITY OF DETROIT HEALTH OFFICE,

March 28, 1894.

Wm. C. Krauss, M. D., Buffalo, N. Y. : We have no trouble with typhoid fever here, no more cases in a year than would be reasonably expected, and it has never been epidemic.

The excreta and urine of typhoid patients are disinfected at once, and buried, and *never* allowed to run into the sewer.

Will you kindly inform me of the salaries paid the Health Commissioner and health officers in your city, also the number of sanitary inspectors employed by the Department of Health, also salaries paid inspectors ? Please answer by letter, not by printed report, and at earliest convenience.

Yours respectfully,
D. McLEOD, M. D., *Health Commissioner.*

Estimated population, 300,000.

HEALTH DEPARTMENT, CITY OF ERIE,
ERIE, Pa., June 11, 1894.

Dr. Krauss : Until recently the Erie physicians have been very negligent in reporting cases of typhoid fever, so we have no authentic record of the number of cases which have existed in the city. In the year 1893 there were but fourteen deaths in the city from that cause. Any additional information will be cheerfully furnished.

Yours very truly,

H. E. FLINT, M. D., *Health Officer.*

Estimated population, 40,000 to 50,000.

It is very evident from these letters that typhoid fever cases are not generally reported to the boards of health, and in the City of Cleveland the large ratio of deaths is due, no doubt, to the fact that the recording of these cases is not obligatory. Perhaps the failure of the health officers of Toledo, Sandusky, Dunkirk and Ashtabula to respond to my letters is due to the fact that no record is kept of typhoid fever.

The information thus gained is, therefore, very unsatisfactory, and the objects for which this investigation was undertaken are in part defeated.

The city bacteriologist has, to a considerable extent, aided me in this enquiry by examining the lake water at the inlet pier, and some from the south shore of the lake. On March 16, 1894, two specimens were examined, taken from the inlet pier in the river, which revealed 124 colonies per cubic centimeter, none of which were of the typhoid bacillus. The examination of the lake water never disclosed any germs of typhoid.

*December, 1893*—Number of water examinations, 1; average number of colonies of bacteria to Cc., 477.

*January, 1894*—Number of examinations, 2; average number of colonies of bacteria, 258.

*February*—Number of examinations, 2; average number of colonies of bacteria to Cc., 184.

*March*—Number of water examinations, Niagara, 5; average number colonies to Cc., 203; Bird Island inlet, 4; average number colonies to Cc., 26,499.

The typhoid bacillus was isolated from Bird Island inlet water from sample taken March 16, 1894.

*April*—Number of water examinations, 5 (two samples being taken from reservoir); the average number of colonies to Cc., 252.

Special examination for typhoid gave negative results.

*May*—Number of examinations, 5 (two being from reservoir and one from pumping station); average number of colonies of bacteria to Cc., 164.

All examinations for typhoid gave negative results.

*June*—Number of water examinations, 14 (four from reservoir, two from pumping station, three from 46 Delaware avenue, five from Lafayette square); the average number of colonies to cc., 206.

All examinations for typhoid gave negative results.

*July*—Number of examinations, 19 (seven from 46 Delaware avenue, three from pumping station, nine from reservoir) ; average number of colonies to cc., 202.

All samples examined for typhoid gave negative results.

*August.*—Number of examinations, 26 ; ten from 46 Delaware avenue, twelve from reservoir, four from pumping station) ; the average number of colonies of bacteria to cc., about 200.

All examinations for typhoid gave negative results.

*September*—Number of examinations 22 (nine from 46 Delaware avenue, eight from reservoir, five from pumping station) ; average number of colonies of bacteria to cc., about 232.

All examinations for typhoid gave negative results.

These results are highly satisfactory and gratifying, but let us not forget that eternal vigilance is the price of safety. Who can tell at what moment an epidemic may break out in one of the lake cities, and the typhoid germs carried along with the lake currents to the inlet pier and pumped into the water mains of this city? The danger may not be great or alarming in this year of grace, 1894, but in 1904 or 1914, if the lake cities increase in population as they have during the last decade or two, and nothing done to prevent the pollution of Lake Erie—by sewage, garbage, street sweepings, etc.,—the immaculate purity of Niagara water will be apt to suffer and along with it the cities on the banks of the Niagara river, which, perhaps, by that time will bear the name of greater Buffalo.

---

(3) Pumps, wells and privy vaults by John H. Pryor, M. D., *chairman of sub-committee.*

Your sub-committee on wells and privy vaults was instructed to collect information concerning the number of wells and privy vaults in the city and their location. An investigation with that object was carried on for some months with the aid of the city authorities. Arrangements were made by which the police department reported by precincts the number and location of privy vaults existing in each and the street department of the Board of Public Works reported the number and location of wells. The investigating agents were the patrolmen, who reported to the captain of the precinct and the foremen of gangs of garbage collectors. When the reports were received by the superintendent of police and the superintendent of streets, they were forwarded to the health commissioner and later transmitted by him to the committee with a communication included in this report. In regard to the report as to the number of wells, it must be said that the information is undoubtedly incomplete and only approximately correct. It is valuable, however, in that the location of a large number is known. The great proportion of privy vaults and wells are confined to a district bounded by Seneca, Michigan and Ferry streets and Fillmore avenue.

Since January, 1892, a large number of privy vaults has been abolished and the effort to remove them is exerted whenever practicable or just. When the privy vault becomes a nuisance it is reported and abated. This investigation has furnished the health department with valuable information, from which deductions may be drawn to guide future action and the effects of privy vaults upon public health may be carefully studied.

It is doubtful whether many of them, especially the sealed vault, should be declared a nuisance. The possibility of the contamination of the water supply in certain localities cannot be considered now, as the returns from the police department do not contain remarks on sanitary condition or surroundings.

When the privy vault cannot be condemned as a nuisance, the Board of Health in certain instances is not justified in closing the same.

Among the poor and in unsewered regions radical measures would cause unnecessary hardship and give rise to reasonable objections. After careful inquiry relating to the action of the Board of Health in this matter and consideration of the dangers of contradistinction to individual rights, your committee refrains from making any suggestions and views. The progress made by the Board of Health is most commendable. Time, the extension of municipal improvements and careful sanitary supervision will gradually and safely obviate this possible source of infection. The presence of a large number of public and private wells in this city must be regarded as dangerous and a constant menace to the public health. Many so-called private wells are not so in fact. Instances are known where several families get their water supply from the same well. Fortunately public pumps are almost a thing of the past. Only a few remain and they should be closed and undoubtedly will be as soon as evidence can be furnished upon which to condemn them. During the past two years the examination of many specimens of well water has demonstrated that over one-half of them are bad and many others are recorded as on the danger line. Repeated examinations often show that the amount of contamination increases and gradually the well water called "fair" becomes "bad."

The plan pursued by the Board of Health is as follows: Chemical and bacteriological examinations of the water are made, and if the result shows sufficient contamination the well is condemned and closed. In this way the number of wells is constantly being diminished. Your committee learns that attempts at progress in this direction are met by determined opposition, and the power of the Board of Health to interfere with alleged private rights is often questioned and combated. The question is not alone, Shall any person be allowed to drink water which has been pronounced detrimental to health, but, Shall a supply of contaminated water which may be used for drinking purposes exist? We are concerned with the cause of infection and its annihilation. Pure water can be obtained, and individuals who prefer drinking unhealthful water should be deprived of the dangerous privilege by stopping the supply. It will be remembered that the vast proportion of wells in this city is located in a district supplied by Niagara river water. At present frequent chemical and bacteriological examinations of the water supply are a constant safeguard against infection and a guaranty of its purity. This intelligent supervision cannot be properly extended to numerous wells and, therefore, private and general protection must be inadequate. It is established as a sanitary fact that wells in a city are a constant source of danger and one that is most easily obviated. Their existence in large numbers encourages the development of infectious foci, which inevitably escape the vigilance of the health department until their character is made manifest by illness.

The water in a private well may be wholesome today, suspicious tomorrow and poisonous the next. Wells must be regarded as easily contaminated and a possible source of infection, and the reason considered why in most instances they should not be immediately abolished. In answer it may be stated that the water may be pure and no other water supply convenient. Such cases exist, and the only wise plan to pursue, it seems, will be to keep them under surveillance and allow them to exist while safe and necessary. In regard to those pronounced wholesome and in use, where the city supply may be obtained, there appears to be two methods which may be pursued. First, a policy may be adopted requiring frequent examinations and tolerance while below the danger limit. Second, condemnation and closure; in other words, the supply abolished. One will secure partial prevention and the other complete. The profession desires and will cordially support a crusade against wells and their increase or introduction in those districts supplied by Niagara river water should be discouraged. Another and more systematic and comprehensive investigation should be made and a complete list of wells and their location obtained by the health department. The water from each should be carefully and frequently examined and wherever consistent with judgment they should be abolished immediately.

Part of the labors of the committee has been to ascertain the cause of the occurrence of epidemics and sporadic cases of typhoid fever in this city. Your sub-committee would report that no agency appears where so direct and tangible an influence in the causation of endemic and sporadic cases of that disease as infected well water. There is ground for the belief that an investigation of the relation between the contaminated well water and isolated cases of typhoid fever would furnish important facts to uphold that statement. Quite recently striking evidence of the part played by infected well water in the causation of typhoid fever has been collected. The facts relating to the cause of the Orange street outbreaks of typhoid fever are so interesting and appropriate that they are included in this report.

Department of Health, Office Health Commissioner,
Buffalo, N. Y., October 6, 1894.

*Dr. John H. Pryor, Buffalo, N. Y.:*

Dear Doctor: I have caused an inspection to be made of the premises No. 114 Orange street, and the inspector reports as follows:

"Upon inspection I find that five of John Dannhauser's children have typhoid fever out of the nine members of the family. One of these is a daughter, who lives at 407 Madison street. All the members of this family drank of the water from the well, although the father and two of the sons did not drink over a glassful of the water, and these three, with the mother, are the ones who are not sick. There is also a young man, by the name of Paul Derringer, who boarded with the Dannhausers, and who drank the water, and now has typhoid and is at the Buffalo General Hospital. At 112 Orange street lives Geo. Finsen, and out of the twelve members of his family six drank the water out of the well at 114 Orange street, and three of these, who drank the water, are now sick with typhoid fever. None of the six who did not partake of the water are sick. In conclusion, out of the sixteen who drank the water from the well upon 114 Orange street, nine now have typhoid fever. These sixteen people are the only ones in the neighborhood who drank the water out of the well, as far as I could ascertain."

August 28, 1894.

Yours very respectfully,

ERNEST WENDE, *Health Commissioner.*

August 25, 1894.

Sample collected from well on premises No. 114 Orange street revealed the Eberth's bacillus typhosus—same responding to all recognized tests. W. G. BISSELL, M. D.

The following communication from the health commissioner contains the results in figures of the investigation.

The list of pumps and wells and their location is embodied in the report, and the report showing the location of privy vaults by precincts is on file in the office of the Board of Health. In conclusion your committee desires to express gratitude for the assistance rendered by the health commissioner, superintendent of police and the Board of Public Works.

DEPARTMENT OF HEALTH, OFFICE HEALTH COMMISSIONER,
BUFFALO, N. Y., September 5, 1894.

*John H. Pryor, M. D., 253 Allen street, Buffalo, N. Y.:*

Dear Sir: In compliance with your request, I beg to inform you that the number of privy vaults reported to this department is 14,199.

Since January, 1892, we have abated 4,845 vaults in this city, and I think we can safely add another 1.000 which have been abated without having been notified by us. The number reported to us as above has been greatly reduced, as some of these were reported early last Spring.

The number of wells reported to us is 235 and 18 Jubilee wells. This number, we believe, is far below the actual number in existence.

Trusting this will be satisfactory, I am, yours respectfully,

ERNEST WENDE, *Health Commissioner.*

The report of the sub-committee was adopted, together with the recommendation that all wells located in districts supplied by Niagara river water should be closed as soon as possible.

(4) Ice supply, by H. E. HAYD, M. D., *chairman of sub-committee.*

The ice supply of the City of Buffalo consists of artificial ice and natural ice. During the last few years a number of artificial ice plants has been developed in the city, but the product is principally used by the manufacturers themselves, although the Arctic Ice company delivers ice to the residents of the east side of Main street and more especially in the immediate neighborhood of their plant. Artificial ice, if properly prepared, is an ideal product. The water must be sterilized and filtered and every precaution taken against subsequent contamination, or germs probably will be found. It is clear, clean and dense, and its density is due to the fact that little or no air enters into its composition. It is much heavier than natural ice. Its refrigerating powers are less, because natural ice melts more easily and faster ; and in this connection it may be said that the Armour Meat Packing company has experimented with artificial ice and found that when it was used in their meat boxes, the temperature would not fall lower than 42° F., while with the natural ice it fell to 38° F. The samples of artificial ice examined in our health department were not absolutely pure, but contained quite a large colony of bacteria, which is no doubt due to the fact that absolute sterilization is a very difficult matter. Not only must the water be sterilized and filtered, but the cans and hands of the workmen, to insure a product without germ life.

However, of all the samples of ice, natural and artificial, examined by Dr. Bissell, of the health department, that of the Arctic Ice company contained the fewest number of bacteria per cubic centimeter.

Of the natural ice about 100,000 tons are used annually in the City of Buffalo, and it is obtained from Lime lake, Silver lake, Chautauqua lake, Cassadaga lake, Lake Erie, East Concord pond, Burlington bay, and Lake Simcoe, Erie canal, Erie basin and Black Rock harbor.

In good ice-producing weather Lake Erie yields a large amount of ice, but owing to our warm winters this source of supply cannot be depended upon. Most of our ice dealers have erected large storage houses on the banks of the interior lakes and get most of their ice from these sources. However, about 8,000 tons of ice was collected in the Lake Erie basin, canal and Black Rock harbor last winter. Of necessity, that collected from the Erie basin, canal and Black Rock harbor must be a dangerous article, since it is contaminated by our outlet sewer as well as the sewage from the South

Buffalo district, which flows down the Buffalo creek. The ice dealers found that ice collected in the basin and harbor was not a very saleable article, because it was vitiated by the oil from the oil refineries which runs into the Buffalo creek.

Ice collected in the lake outside of the breakwater and in the neighborhood of the light house, is a clear and reasonably pure product, but the expense of procuring it, as well as the uncertainty of our winter weather, renders this site impracticable. However, the Buffalo Ice company and L. B. Banks & Co. cut considerable ice in this neighborhood last winter. The Buffalo Ice company cut 400 to 500 tons at the foot of Genesee street and 3,000 tons outside the breakwater, and L. B. Banks & Co. cut in the Coit slip about 500 tons—between the D., L. & W. railroad coal sluice and the N. Y. C. railroad coal docks—and about 1,500 outside the breakwater. However, the ice from inside the breakwater, I am assured by these gentlemen, was used simply to fill the lower tiers of their ice houses and for refrigeration purposes only. Ice was also collected in the Black Rock harbor and in the Erie canal, perhaps 1,500 to 2,000 tons, and I am informed that such ice has been sold for ordinary domestic purposes, notwithstanding the fact that these places were proven last winter to be polluted quarters.

Ice, in order to be an ideal product, must be obtained from a pure and rapidly flowing stream,—in fact, a current of four miles an hour produces the best ice. The temperature must fall rapidly below the freezing point and remain there for some time. Small ponds and quiet bodies of water do not yield fine ice, nor is the ice safe when the water contains a net amount of animal and vegetable matter. The ice used in Berlin, according to Sternberg, is collected from the surface of the lakes and rivers in the vicinity of the city and contained from a few hundred to 25,000 bacteria per cubic centimeter.

Hudson river ice, according to Prudden, six miles from Albany, had an average of 398 bacteria per centimeter, while snow ice or superficial ice had 9,187. Ice lower down the river contained an average of 189 in the transparent ice or ice from the river, and 3,693 in the snow ice. In ice as in water it may be said "that an excessive amount of bacteria is an indication that the water contains a large amount of organic water which serves as a pabulum for microörganisms, and although wet ice or water may be potable, yet Sternberg says that water with more than 500 bacteria to the cubic centimeter may be harmless, it is still looked upon as suspicious, and water containing 1,000 bacteria or more is presumably contaminated with sewage and surface drainage and should be rejected or filtered before it is fit for drinking purposes, and I presume the same deductions may be drawn in reference to ice.

Silver Lake is situated in Wyoming County and is about one-half mile wide and four miles long. It is fed by springs entirely, except in the fall and spring, at which time it receives the surface water from the surrounding hills, but is limited as its location is 1,200 feet above that of Lake Ontario. A small creek which flows for a mile or two also empties into the lake. Its banks are clean and dry on both sides and the cottages and enclosures on the lake are well cared for, as the night soil is carefully disposed of. No sewers empty into the lake and only a very few tile drains. For two months in the year the cottages are occupied. The outflow of the lake is 900 cubic feet per minute, and together with the overflow which occurs each fall and spring renders the water subject to healthful change most of the time. The storage ice capacity is 30,000 tons, most of which comes to Buffalo.

Lime Lake is located in Cattaraugus County on the Cattaraugus Hills and is 1,150 feet higher than Lake Erie. It is one mile long, one-half mile wide and is from 40 to 100 feet deep. It is fed by natural springs and has an outlet. The current is pretty strong, and the water is thus changed. There is located on the bank the Cattaraugus County House and a grove and summer hotel. No sewers empty into it and the sewage of the County House is directed away from the lake and disposed of. The ice storage capacity is 100,000 tons, of which 50,000 comes to Buffalo.

Chautauqua Lake supplies Buffalo with a great deal of ice and more particularly last year, as the yield from Lake Erie was very small,—in fact, all our ice dealers have bought and sold Chautauqua ice this year. There are located on the lake three large ice houses, the largest at Mayville with a capacity of 50,000 tons. Another is located close by and a third at Lakewood. The ice from Chautauqua Lake, as seen by the health department's report, is of excellent quality notwithstanding the fact that so many cottages are on the lake.

Cassadaga Lake supplies about 10,000 tons and is principally sold by the Queen City Ice Co.

Burlington Bay last year gave Buffalo about 8,000 to 10,000 tons and from the report made by the health department it is a very dangerous product and should be excluded from our city altogether.

From Orillia and Barry on Lake Simcoe a small quantity of ice comes and it is of very excellent quality.

Five hundred tons were cut in the Erie canal at Ferry street by Austin McTigue and about 2,000 in Black Rock harbor.

The East Concord pond on the B., R. & P. railroad supplies J. P. Sullivan with about 8,000 to 10,000 tons. The bacteriological examination shows this ice to be heavily charged with vegetable life, and perhaps to a dangerous extent.

I append herewith a report from the health department which shows the number of colonies of bacteria in each sample of ice examined in their office.

DEPARTMENT OF HEALTH, OFFICE HEALTH COMMISSIONER,
BACTERIOLOGICAL LABORATORY,
BUFFALO, N. Y., October 20, 1894.

*Ernest Wende, M. D., Health Commissioner, Buffalo, N. Y.:*

Dear Sir: I have the honor to submit the following report of the bacteriological examinations of samples of ice collected by me October 9, 1894, said to have been derived from the following-named sources and supplied to the City of Buffalo.

I consider the test a fair one, in that nutrient gelatine-agar media was used, it being a fact that the proportionate number of colonies of bacteria which develop in water, and necessarily ice, examinations is much greater where gelatine media alone is used as compared with agar.

Petri dish method—nutrient gelatine-agar media—eight days' count, at room temperature, showing the average number of colonies of bacteria to the cubic centimeter:

Buffalo Ice Co., Silver Lake ice, Builders' Exchange: Lime Lake, 272; Silver Lake, 240; Erie basin, out; Lake Erie, out; Chautauqua Lake, out.

Queen City Ice Co., W. L. Markham, Manager, 159 Pearl street: Hamilton, Ont., 364; Haliburton, Ont., out; Cassadaga Lake, 261; on fifth day, dish accidentally broken.

American Ice Co., 500 Louisiana street: Lime Lake (usual source), out; Chautauqua Lake, 164; Cassadaga Lake, 284; have bought Arctic ice, out.

L. B. Banks & Co., 219 Erie street: Coit slip—simply cut a channel through the ice in this slip to get to the lake—do not use it; Lime Lake, 492; Lake Erie, 316; Hamilton, Ont., out; Chautauqua Lake, out. Propose to cut from Grand River, Dunville, Ont.

Rebstock & Blust, 192 Grace street: Black Rock harbor, out. Buying at present from the American Ice Co. and the Buffalo Ice Co., the ice used being the same as their supply.

T. J. Nunan Ice Co., 477 Seneca street: Hamilton, Ont., out; Chautauqua Lake, dish destroyed; Lime Lake, 576.

E. Webster, Son & Co., 308 Main street: Lime Lake, 468; Chautauqua Lake, out. Have bought Arctic ice. Propose to use mostly Arctic ice in the future.

Arctic Ice & Storage Co., Fillmore avenue: Artificial ice manufactured by themselves, 116.

Niagara Ice Co., W. W. Webster, 849 and 851 Seventh street: Buys of Banks & Co.; supplies the same ice as they obtain.

Austin McTigue, 96 West Ferry street: Out of business; had about 200 tons; sold this to Rebstock & Blust. Ice came from Buffalo harbor, foot of Ferry street, out.

Jacob Dold Packing Co., William street: Manufactured, 180.

J. P. Sullivan, Excelsior Ice Co., 110 Chicago street: Buying ice at present from American Ice Co.; East Concord pond, out.

A qualitative examination was made of each sample as to the presence of Eberth's bacillus typhosus, by Paretti method, each examination giving negative results.

Most respectfully yours,

(Signed) WM. G. BISSELL.

From this report it will be seen that Buffalo is to be congratulated in having such an excellent ice supply, and its dealers have shown business sagacity in locating their ice houses where a reasonably pure ice can always be obtained. Of all the samples examined only that from Burlington Bay, Hamilton, Ont., could be condemned upon the grounds of having a dangerous quantity of bacteria per cubic centimeter.

Your committee makes the following recommendations:

1. That the Board of Health be requested to make regular stated examinations of ice from all the sources of supply to the City of Buffalo, and that all found to contain bacteria in excess of the recognized limit of safety be condemned.

2 That ice from Burlington Bay be excluded from the city.

3. That the ice collected in the Erie Basin, Erie canal and Black Rock harbor be condemned and should not be used at all, unless it can be proven to the satisfaction of the health department that such ice is never used for potable purposes, or for any domestic purposes, where there may be the slightest possibility of contaminating the food supply.

4. All ice hereafter brought into the city is to be allowed only after the Board of Health has been satisfied that the water of such place and the location of the storage houses are safe.

5. Your committee also recommends that artificial ice, properly prepared, be used for all domestic purposes.

---

(5) Milk supply, by Henry R. Hopkins, M. D., *chairman of sub-committee.*

*Presented to the Academy May 14, 1895.*

Mr. President—Your committee on the milk supply of the City of Buffalo would respectfully report:

The state board of health divides the state into eight sanitary districts, following county lines; this is for the purpose of observation, record and study of vital statistics.

Of these districts the Maritime includes the counties of Westchester, New York, Richmond, Kings, Queens and Suffolk—has a population of over three and a half millions, of which over 87 per cent. live in cities.

The seventh, known as the West Central district, includes the counties Cayuga, Tompkins, Seneca, Schuyler, Ontario, Yates, Livingston, Genesee and Wyoming—has a population of over 311,000, of which over 12 per cent. live in cities.

The eighth sanitary district is known as the Lake Ontario and Western, includes the counties Oswego, Wayne, Monroe, Orleans, Niagara and Erie—has a population of over 761,000, of which over 60 per cent. live in cities.

The last report of the state board of health is its thirteenth report. Study of these reveals, with other things, that the mortality of our people is constantly and uniformly influenced by environment and by season. The death-rate of those dwelling in cities is always larger than those living in the country, and this is true in a more particular degree of those under five years of age.

The thirteenth report of the board shows a mortality in the Maritime district of 75,444, being at the rate of 23.37 per 1,000. Of these deaths 33,087 were under five years of age, being 41.2 of the whole number of deaths.

In the West Central district, the whole number of deaths was 4,445 at the rate of 14.29 per 1,000. Of these 561 were under five years of age, being 12.6 of the whole number of deaths.

In the Lake Ontario and Western district, whole number of deaths was 13,024, at the rate of 17.10 per 1,000. Of these 4,346 were under five years of age, being 33.4 of the total deaths.

In answer to an inquiry at the department of health of Buffalo, we learn that during the year ending September 30, 1894, there were in our city 5,520 deaths. Of this number 2,278 were under five years of age, being 41.26 of the total deaths.

Of the deaths under five years over 39.100 occurred in the three months of July, August and September.

No more important question can claim the attention of the medical profession, or the attention of the general public, than this matter of our enormous death-rate of those under five years of age —this slaughter of innocents—going on from year to year, which rises with each summer to the proportions of a wholesale massacre.

We learn from United States Census Bulletin, No. 378, issued March 19, 1894, that there were on farms and in stables in the United States, June 1, 1890, 16,511,950 milch cows, and that these cows produced during the previous year 5,209,125,567 gallons of milk—315.48 gallons to each milch cow and 83.18 gallons to each inhabitant of the country. The country, in fact, literally flows with milk and honey—milk, the kindly fruit of the earth, and honey, the typical reward of industry.

Medical men are slowly but surely reaching the conviction that the cause of the alarmingly high death-rate of those under five years, who live in cities, as compared with those who live in the country, is to be laid at the door of the milk supply of cities—to the ignorant, careless and fraudulent adulteration of city milk— and public opinion, the result of patient and persevering medical teaching, is aroused, and from time to time makes itself felt in action, in ordinances of the municipality and again in statutes of the commonwealth.

The purpose of this report is to again place on record the convictions of the individual members of the Academy of Medicine, as to the vital importance to our city and state of the lives of our young children, to again call attention of those in authority, and more particularly of those charged with the responsibility of executing our health ordinances and health statutes, to the alarmingly high death-rate of our city dwellers under five years of age, especially during the months of summer.

And just here it may not be out of order to pause a moment to remark the singularly fortunate location of our city as regards the immediate contiguity to the hills and valleys, the abundant pastures, the endless streams of cool and pure water, the countless farms and dairies of the finest grazing region known and farmed over the entire world— which lies at our very door.

This matchless suburban country surrounding our city in all directions, save our lake front, is penetrated in all parts with an unrivaled system of railroads with well-appointed trains, thus placing each home in our city within an hour's time of some farm or dairy.

Your committee is of the opinion that the high mortality of those under five years of age in Buffalo is not the fault of its location or environments, but must be brought to the doors of the medical profession, whose duty it is to see that public opinion is kept alive and efficient, to the end that all care and diligence be exercised to protect the helpless from selfish, greedy and criminal adulteration of our milk supply.

It is never a pleasant duty to direct attention to the shortcomings of any class of our citizens, and more particularly so when the error involves a question of bad morals, but the lesson of the history of the dairy commission of the State of New York is the lesson of all attempts to supervise and study this question, viz.: that trade instinct may not be trusted to deal honestly in the milk business. State or municipal supervision is absolutely essential to protect the consumer against dishonest adulteration.

The result of systematic examination of milkman's milk by our state authorities, resulted in the arrest of sixty-two milk peddlers doing business in this city in a single season for selling adulterated milk. This is simply an illustration of how trade methods work in the milk supply business. Unquestionably these notorious sixty-two were not the first or the last of their kind.

The reasons for the need of state or other care in the supervision of milk supply naturally fall into two groups—(1) the consumers ; (2) the product.

Concerning the consumers it must suffice to observe that children, and especially those under five years of age, are particularly liable to diseases of various kinds, and that this law of liability increases or intensifies with the youth of the child, and that it is worthy of particular remembrance that as the susceptibility or proclivity of the child increases so the habit becomes general that milk is almost the sole food. Thus two important lines, susceptibility or proclivity to disease and exclusive milk diet, meet and run side by side in our infants and young children and the resultant appears in the enormous death-rate shown in the mortality statistics of our cities of the class reached—those under five years of age.

Your committee is quite conscious that the foregoing is by no means an adequate statement of the topic, but feels the importance of brevity, that a report should not aim to be a treatise.

We may profitably divide the considerations of the question of city milk and food product into two heads, which we may designate (1) commercial, and (2) professional or scientific. Under the first may be considered the facts which the common observation of lay people have shown to be essential, to the end that milk may be the food that Nature seems to have intended it to be. Such observation long ago established the conviction that healthy milk may only be obtained of healthy cows, grazing at will in well-shaded and abundantly-watered pastures during the months of warm weather, and warmly housed in clean, well-lighted and ventilated stables during the cold months, regularly fed with well-cured hay or fodder, provided with abundant drink of pure water, and also given liberty daily to exercise at will in a suitable yard.

Common observation also abundantly attests that wholesome milk may only be milked from cows that are clean by a milker also clean; that pollution of milk, rendering it unfit for use, frequently takes place during the milking, by dirt falling from the cow or the milker into the pail.

To make these precautions effective—and all must admit that they are most fundamental and essential—constant supervision and frequent inspection of all farms, stables and herds engaged in city trade is requisite. How formidable a task it is to introduce Listerism into the milk trade of our cities we can get a hint by recalling the effort and agitation and time required to introduce Listerism into our surgeries and lying-in rooms. Howbeit, the never-ending slaughter of our innocents demands of the medical profession the accomplishment of this result. Indications were never more plain and the destructive effects of carelessness and ignorance never more apparent.

Common observation also teaches that milk is a most delicate and sensitive product, requiring most intelligent and careful handling in order to retain its innocence and virtues, its value as a food.

To keep milk free from contamination, from the milking to its delivery to the consumer, is absolutely essential to its wholesomeness; but this is not enough. Milk must be allowed to cool promptly, and this requires special treatment. Freshly drawn milk contains a peculiar volatile constituent, which is both offensive to taste and prejudicial to keeping. This must be allowed to pass off, and it is fortunate for the farmer that the processes of cooling and airing may go on at the same time—the conditions favoring one are also favorable to the other.

Listerism of our milk supply also demands that the clean, pure milk, well cooled and aired, shall be placed in absolutely clean cans for transportation to the city ; the sensitiveness and delicacy of the product requires that the hauling to the railroad station be done under suitable conditions, that while waiting at the station for the train that undue exposure to heat or cold be avoided, that conditions of cleanliness and suitable temperature attend the milk during the entire journey from the farm to the ice box of the consumer.

In order to secure the handling and carriage of milk as indicated, there must be, in addition to the inspection and supervision already referred to, the habitual inspection of all vehicles used in the transportation of milk—cars, wagons or carts—backed by the proper authority, to see that the desired results are obtained. Not, as is too frequently the case, that there be either open disregard of law and ordinance, or at least, or at best, only such formal and perfunctory compliance therewith as to fail utterly to accomplish the benign and wise intentions of our law-makers.

Up to this point your attention has been directed to losses or injuries liable to befall city milk, the result of ignorance or carelessness on the part of producers, handlers or dealers ; but we may not close this topic without reference to the sadly inferior quality of milk received by the city consumer, as compared with the well-known uniform characteristics of that product as discovered by examination at its home in the country. It is sufficient to say that systematic testing of city milk by representatives of state or municipality, duly authorised and qualified, has demonstrated over and over again that wholesale watering or other adulteration of milk while passing through the hands of milk dealers was the custom. That such was the case has been the common knowledge of the medical profession and of the laity for generations. In this state the facts were judicially determined and officially announced by the work and reports of our dairy commission created by act of the legislature of April 24, 1884.

Your committee has given the laws of the state and the ordinances of our chief cities and of our city bearing upon this matter due study and consideration, and from this study heartily congratulates the public upon the intelligence and earnest spirit of appreciation shown by our law-makers, that it is not too much to say that our statutes and ordinances have only to be properly enforced to secure to our people who dwell in cities the inestimable advantage of a pure milk supply.

How thoroughly in earnest the Empire State is, we get an idea by a few brief quotations from the law of April 24, 1884, entitled, An act to prevent deception in sales of dairy products :

> No person or persons shall sell or exchange, or expose for sale or exchange, any unclean, impure, unhealthy, adulterated or unwholesome milk, or shall offer for sale any article of food made from the same or of cream from the same.

Violation of this section is made a misdemeanor punishable by fine from $25 to $200 or by imprisonment from one to six months, or by both fine and imprisonment.

> No person shall keep cows for the production of milk for the market, or for sale or exchange, or for manufacturing the same, or cream from the same into articles of food in a crowded or unhealthy condition, or feed the cows on food that is unhealthy or that produces impure, unhealthy, diseased or unwholesome milk.

Penalties for the violation of this section are named like those just mentioned.

The execution of this law is placed in the hands of an officer to be known as the New York state dairy commissioner, who is authorized to appoint an assistant commissioner and to employ such experts, chemists, agents and counsel as may be deemed by him necessary.

The sum of $30,000 was appropriated for the purposes of this work, and it was ordered that the commissioner and his agents shall have full access, egress and ingress to all places of business, factories, farms, buildings, carriages, cars, vessels and cans used in the manufacture and sale of dairy products or any imitation thereof ; with authority to open any package, can or vessel containing such articles which may be manufactured, sold or exposed for sale in violation of the provisions of this act, and may inspect the contents therein and may take therefrom samples for analysis.

This law further provided that " The doing of anything prohibited being done, and the not doing of anything directed to be done in this act shall be presumptive evidence of a willful intent to violate the different sections and provisions hereof."

The annual reports of the dairy commissioner revealed a state of facts in the milk supply of our cities amply justifying the expenditure of money—and the policy of the state as indicated by the law of 1884 has steadily grown along the same lines.

Year by year the laws have been reënacted, the powers of the commission have been extended, the appropriation for expenses has been increased to $50,000 per year, the dairy commission has been raised to the dignity and importance of the department of agriculture, and the importance to the public health of a pure milk supply is fully realized, and provision made for that constant supervision and inspection which experience has demonstrated to be indispensable.

Your committee has read and studied the reports of the local agents of the dairy commission—now the department of agriculture—and has conferred with the department chemist, Prof. Miller, and from these several sources receives the same impression—that the uniform condition of our milk supply has been greatly improved; that dilutions and adulterations of milk while in the hands of dealers are now as rare as before they were common, and that this improvement is due to state inspection and to the wholesome punishment of the offenders.

Here it may be noted that the total number of annual deaths in the state last reported, under five years of age, was 41.643. We have seen that of these deaths a large proportion takes place during the three hot months and believe that two-thirds of this number are deaths from preventable diseases, and that in this work of prevention a clean, pure milk supply is an important factor.

With these observations we conclude the considerations of our milk supply from the commercial or lay view point and pass to that more entirely professional in quality.

The consideration of the milk supply from the more professional side brings before us facts of the highest interest and scientific character.

Perhaps it is not too much to say that no discovery of modern times is more potential for the good of man than the finding of the pioneers of scientific medicine that our domestic animals show their near relationship to us in structure and composition by showing a large number of our diseases, and certainly it may be affirmed that medicine today presents no more inviting field for study than the question of the relation of the diseases of the food-bearing animals to the morbility and mortality of man.

The history of medical thought on this subject furnishes an apt illustration of the superiority and priority of the art over the science of medicine, and at the same time and with the same facts also shows how composite and many-sided is the human mind, and how much stronger claim for popular confidence has medicine which gives evidence that it has not only the motive and inspiration of art but the accuracy and thoroughness of detail of science.

The art of medicine has long known that the milk of the cow, the mare, or the mother, might be rendered injurious or poisonous to the offspring or the consumer by reason of unfavorable bodily conditions or mental state on the part of the milk-giver, and the science of medicine is just beginning to learn something of the why and how of this interesting phenomena.

Modern writers teach with positiveness and confidence that the milk of the cow is liable to become the bearer of the infection of the following diseases, known to prevail among our herds: eczema contagiosa, pleura-pneumonia, scarlatina, diphtheria, pyemia, septicemia, milk-sickness or trembles, anthrax, actinomycosis and tuberculosis.

Formidable as is this list, it by no means exhausts the number of the communicable diseases, contagious or infectious, to which the bovine species is subject; and when you extend this thought and include the fact that cats, dogs, fowls, pigs, goats, sheep and horses are in like manner subject to these diseases, or many of them, you get a hint of the practical importance to health, personal or public, of the diseases of the lower animals communicable to man.

Your committee does not propose to trespass upon the endurance of the Academy by attempting to review the rapidly increasing literature bearing upon this entire question, but will confine the consideration to a single disease—tuberculosis; and in the treatment of this topic will take it for granted that tuberculosis is a communicable disease, spreading from man to man, from man to many of the domestic animals, notably the milk cow, and likewise spreading from the domestic animals to man.

That tuberculosis prevails among our herds to an extent not dreamed of, and is almost as important a factor in increasing the losses and expenses of the former as it is in the causation of suffering and death of the human family is an admitted fact.

History seems likely to note Koch's tuberculin as his chief discovery, and has already given tuberculin its position in medicine as a reliable test for the recognition of otherwise latent tuberculosis of the domestic animals. The accuracy and reliability of the tuberculin reaction in the presence of incipient, mild or latent tuberculosis is uniform, and gives the test an importance which can hardly be over-estimated or over-stated.

Your committee is pained to note the over-conservatism of certain medical investigators of this important matter, particularly those of continental Europe, who have hesitated to give scientific authority to the condemnation of animals suffering from slight or localized tuberculosis, holding that the flesh of such might safely be used for food ; and, in the case of cows, holding that unless the disease was general, or that the udder was its seat, the milk might be used with safety.

After a careful study of the literature upon this matter, your committee does not hesitate to report that the above-noted opinion is not in accord with the great mass of testimony bearing upon the question ; that such conservatism is unwise, and its proposed economy, in effect, will be found wasteful and extravagant.

It would seem that on this point sentiment-taste should be allowed to dictate, and we may safely trust intelligent sentiment to dictate that neither milk nor any product of milk, or meat or any food product of any kind whatsoever, shall be taken from any animal suffering from tuberculosis, and that the tuberculin reaction may be relied upon to determine the presence of the disease, otherwise not demonstrable.

And it would also seem that, ultimately, sentiment and science are to be found in accord on this point as upon most important matters, and we may confidently rely upon investigation ultimately furnishing the reason why food products from animals suffering from tuberculosis are as inimical to public health, as they have always been offensive to intelligent taste.

In connection with this, attention should be called to the argument of James Law, of the faculty of Cornell, in Bulletin No. 65, April, 1894, entitled Tuberculosis in Relation to Animal Industry and Public Health. The entire question is presented there in a thorough and able manner.

This argument does not assume to present the results of any startling discovery or in the main to be based upon new work, but by an intelligent handling of the facts and truths of bacteriology, not subject to dispute, by a wise adjustment of the emphasis appropriate to the various bits of our knowledge, succeeds in establishing conclusions of the most important and practical character.

Law directs attention to the fact of the two modes of bacterial disease—bacterial invasion and bacterial intoxication—anthrax an instance of the former and diphtheria of the latter. That in anthrax, as in all the bacterial diseases of the first class, the organism was inimical to its host by virtue of its ability to increase and multiply rapidly in the blood and other fluids of the body, to literally swarm in every part of the body, to choke the blood-vessels and thereby rob the tissues and organs of nutrition and also to devour the nutrients of the body, more particularly the bearers of oxygen, and in this two-fold manner prevent the normal nutrition of important parts or of the entire body.

The action or rôle of bacteria in the other class of diseases, known as diseases from bacterial intoxication, differs from the foregoing in important particulars. The method in diphtheria, an instance of disease from bacterial intoxication, gives a good illustration of this class. In this disease it is to be noted that bacteria show a certain effective affinity for given tissues and localities, as the mucous membranes of the throat, mouth, nose and larynx. That once seated in a certain spot the disease shows but slight, if any, tendency to spread, but following the habits of plants, sends down roots into the soil and lifts up branches and leaves upon the surface thereof, but in the main continues at the spot where it would seem to have been planted.

However, as a result or rather as a resultant of the life and growth of the pathogenic bacteria in the tissues, certain chemical substances are produced, which products are soluble in the fluids of the body and thereby pass into the circulation.

These substances called toxines are poisonous—in some instances frightfully poisonous—and their continued elaboration in the system and their continued poisonous effects upon various tissues and organs of the body constitute the essentials of the disease.

Tuberculin is the toxine of tuberculosis, the most important member of the class of diseases produced by bacterial intoxication, the cause of about one-seventh of human morbility and mortality, the most important of the diseases of the domesticated animals and of man and an important factor in the high death-rate of those under five years of age.

The entire rôle of tuberculin in causing and spreading tuberculosis may never be known. Sufficient data are at hand, however, to give to this malign substance a most unenviable notoriety and importance.

But recently, experimenters seemed satisfied to be able to assure us that a given specimen of milk was free from the presence of the bacillus of tuberculosis ; it would now seem that milk may be free from the bacillus and yet hold in solution a sufficient quantity of tuberculin to make it not only unfit for food for animals or man, but possibly the determining cause in spreading the disease, tuberculosis.

This observation has a practical bearing upon the question of the fitness for food of meat taken from any part of the carcass of an animal suffering from tuberculosis in any form or degree, and a singular significance to the question of the healthfulness of the milk of the tuberculosis cow.

Here it is well to note again the unscientific advice of those who, looking too exclusively at the bacillus of tuberculosis and forgetting or failing to give due weight to the tuberculous toxines, recommend the use of meat or milk from the tuberculosis animals, provided the same be cooked, depending upon the heat to kill the bacillus.

How unscientific and unsafe this advice is, we see when we recall that Koch's tuberculin is a sterile product absolutely free from bacilli, but its short-lived use as a therapeutic agent and its longer and longer use as a diagnostic agent, furnish unmistakable proof that the tuberculosis poisons stimulate and quicken tuberculosis processes and that food containing these poisons is unfit for the use of man or beast.

Your committee therefore reports that it cannot concur in the opinion of those who declare that wholesome meat may be found in the carcass of an animal having tuberculosis, or that wholesome milk may be drawn from a cow so diseased, provided that the udder is not the site of the tuberculosis, but holds the other view that the presence of tuberculosis as revealed by the tuberculin test or the inspection of the carcass should be conclusive evidence that such animals are not suitable sources of food, either of meat or of milk.

During the previous year our state made its first attempt to test with tuberculin the health of milch cows under the direction of the State Board of Health. This examination has not been general, but was confined to parts of seventeen counties, and

26,931 animals were tested, of which number 944 were killed. The result of this examination was to disclose the startling fact that in every dairy of twenty-eight cows will be found one with tuberculosis.

The whole number of milch cows reported in the state by the last United States census is 1,552,373, and assuming that the presence of tuberculosis as revealed by our first year's search is a reliable index of its general distribution, we find ourselves confronted with the horrifying probability that during the last year we have consumed the milk of 55,427 tuberculosis cows.

The bearing of this conclusion upon the mortality of those under five years of age obviously needs no elaboration, no argument.

In order to emphasize the more important facts of this subject, the committee submits the following summary:

(1) That the preservation of the public health is the first consideration of an intelligent community, and that the medical profession is the natural source of enlightenment and action in sanitary affairs.

(2) That the death-rate of those under five years of age who live in cities is an impeachment of the competency of our knowledge of sanitary laws, or of our ability to carry such laws to proper execution and effect.

(3) That the problem of the milk supply of cities is of the first importance, touching all who have or hope to have children, and is perhaps the most important factor in the high death-rate of the young.

(4) That truth, light, honesty, purity, life saving action in behalf of our children is demanded here in Buffalo, in spite of the fact that nature has surrounded us with environs of unsurpassed advantages, more particularly regarding the supply of the great foods,—fresh air, clean water and pure sweet milk.

(5) We would heartily congratulate our citizens upon our state laws and the execution of the same, the work of the New York State Dairy Commission—now the Department of Agriculture—also upon the wise and comprehensive character of the health ordinances of our city, and upon the efficient and able Department of Health. The scope and purpose of these laws and ordinances we consider most intelligent. Their proper execution should be our first and highest duty.

(6) We would also invite the attention of all our citizens to the important character of the relation of the farmer and dairyman to the general welfare, the public health ; but more particularly to the high quality of skill, care and fidelity demanded of him in producing milk for sale or exchange, to the end that such distinguished services be regarded properly and also be rewarded suitably.

(7) That Listerism of our milk supply from country farm to city kitchen is imperatively demanded in the interests of public health.

(8) That a wholesome milk supply for Buffalo is only possible as the result of constant inspection and supervision by trained examiners, in the interests and employ of the public, the scope of the examination to include every factor in the case, theoretical or practical.

(9) That it is of first importance to recognize our present knowledge as to the existence of communicable disease among our domestic animals, and that all light possible should be thrown upon the methods of communication of such disease from man to animal, from animal to animal and from animal to man.

(10) That in the case of the health of milch cows, systematic tests for tuberculosis with tuberculin should be made, and all animals suffering from the disease killed and their carcasses burned or otherwise suitably sterilized.

(11) That as soon thereafter as suitable provision is made for such inspection and tests that the department of health be directed to withhold permit for the sale of milk or any product from the same from cows, unless such animals are duly registered by the proper officer as in good health and free from tuberculosis or other communicable disease.

Respectfully submitted,

Chas. G. Stockton, *Chairman*,

P. W. Van Peyma,

W. C. Krauss,

